# Quantifying Inter- and Intra-Population Niche Variability Using Hierarchical Bayesian Stable Isotope Mixing Models

**DOI:** 10.1371/journal.pone.0006187

**Published:** 2009-07-09

**Authors:** Brice X. Semmens, Eric J. Ward, Jonathan W. Moore, Chris T. Darimont

**Affiliations:** 1 Northwest Fisheries Science Center, National Marine Fisheries Service, National Oceanic & Atmospheric Administration, Seattle, Washington, United States of America; 2 Department of Ecology and Evolutionary Biology, University of California Santa Cruz, Santa Cruz, California, United States of America; 3 Environmental Studies Department, University of California Santa Cruz, Santa Cruz, California, United States of America; University of California, Berkeley, United States of America

## Abstract

Variability in resource use defines the width of a trophic niche occupied by a population. Intra-population variability in resource use may occur across hierarchical levels of population structure from individuals to subpopulations. Understanding how levels of population organization contribute to population niche width is critical to ecology and evolution. Here we describe a hierarchical stable isotope mixing model that can simultaneously estimate both the prey composition of a consumer diet and the diet variability among individuals and across levels of population organization. By explicitly estimating variance components for multiple scales, the model can deconstruct the niche width of a consumer population into relevant levels of population structure. We apply this new approach to stable isotope data from a population of gray wolves from coastal British Columbia, and show support for extensive intra-population niche variability among individuals, social groups, and geographically isolated subpopulations. The analytic method we describe improves mixing models by accounting for diet variability, and improves isotope niche width analysis by quantitatively assessing the contribution of levels of organization to the niche width of a population.

## Introduction

The niche concept, which provides a tractable measure of the environment encountered by organisms, figures prominently in ecological and evolutionary theory [1]–[3]. Dimensions related to foraging are often emphasized, following the ‘eat or be eaten’ dictum that unites organisms [4]. Much of the literature anchors the niche to the level of species. However, the niche of a species is the collective response of individuals, groups, and sub-populations to complex ecological and evolutionary processes. Thus, niche differences across relevant levels of population structure collectively comprise a niche of a species or population.

The role of individual variation in shaping a population's niche was first articulated as a component of Van Valen's [5] niche variation hypothesis. Examining the niches of mainland and island birds, Van Valen proposed that population niche width expansion can occur via increased among-individual variation in foraging, such as he observed in island bird populations that were released from interspecific competition. Recently, Bolnick et al. [6] reviewed support for the concept of the ‘individual niche’, and identified evidence from 97 species across a broad range of taxa. In some of these cases, among-individual foraging niche accounted for most of the total population niche width.

Social organization and spatial patterns in resources can yield niche variation at levels above the individual. In social animals, for example, group membership might exert strong influence on diet. Individuals that forage together and encounter the same resources at the same time may have very similar diets, while diets may vary substantially among social groups. On the other hand, social foraging might lead to intense intraspecific competition [7], [8], particularly if dominance hierarchies exist and there are quality differences among prey items [9]. Regardless of sociality, spatial resource heterogeneity can result in differences in the abundance of prey available to consumers. These spatial differences in prey availability likely influence the diets of individuals (e.g. differences in quality among defended territories) or whole groups of individuals (e.g. a sub-population occupying marginal habitat). Indeed, Hutchinson [4] invoked the ‘mosaic nature of the environment’ in his concept of the niche and the causes of its variation.

How can trophic niche variation across multiple scales of population structure be quantified? Elton [2] proposed that the niche was the sum of all interactions, especially trophic, that links an organism to all others. The flow of atoms from resources to consumers can be tracked using measurements of the stable isotope composition of tissue (reviews in [10], [11]). Stable isotope data thus reflect the feeding behaviors of individuals that share otherwise common ecological conditions over long periods, permitting investigations of intra-population variation [12], [13].

There are two general methods for analyzing stable isotope data in trophic ecology that, until now, have remained mutually exclusive. First, stable isotope data have been used to quantify the niche width of consumers [6], [12]–[14] by drawing inference from patterns in isotope variability exclusive of the underlying trophic processes (i.e. the contribution of different prey items to a consumer diet). Second, stable isotope data have been used to explicitly quantify the contribution of prey to consumers using mixing models (e.g., [15], [16]). While the sophistication of mixing models has evolved over the last few decades [15], [17], [18], these models have not incorporated intra-population variability in eating patterns; rather, all mixing models have assumed that the proportional contribution of prey to a consuming population's diet is fixed such that all individuals have identical diets. Clearly, an integrated analytic framework that uses isotope data to estimate both the niche width and diet composition of consumers would reduce the assumptions and improve the performance of the two currently independent methods of analysis.

In this paper we describe a novel analytic framework for using stable isotope data to infer the prey composition of consumer diets while simultaneously estimating variability in diet composition across multiple levels of the consumer's population structure. By explicitly estimating the variance components for multiple scales, the niche width of a consumer population can be deconstructed into relevant levels of population structure. Our modeling approach extends the Bayesian stable isotope mixing model formulation described by Moore and Semmens [15] in three important ways: 1) it is hierarchically structured in order to account for differences in diet across multiple levels of population structure, 2) it incorporates variance in the diet composition of individual consumers, and 3) it uses explicit model comparison to quantify the relative support for the competing models. To demonstrate our approach, we analyze δ^13^C and δ^15^N stable isotope data from a population of coastal gray wolves (*Canis lupus*) with a complex, nested population structure, comprised of 3 subpopulations from different geographic regions, multiple social groups (packs) within the subpopulations, and multiple individuals within groups [14]. The new modeling approaches reveal that variation in feeding habits among subpopulations, social groups, and individuals all contribute substantially to the niche width of wolves.

## Methods

The incorporation of individual diet heterogeneity and/or nestedness into a mixing model presents a non-trivial computational challenge due to the highly constrained covariance structure of diet compositions (i.e. compositions must sum to unity) and the resultant non-normal variance associated with compositional data. Below, we outline two analytic approaches for incorporating diet variability into hierarchical Bayesian stable isotope mixing models.

In order to use these methods, researchers must have the following types of information: 1) The means and variances of the isotopic signatures for all possible prey items (one or more), 2) the means and variances of fractionation for each isotope (one or more) 3) the isotope signatures of individual consumers, and 4) individual assignments to the different levels used in the analysis (e.g. wolf #1 belongs to the 2^nd^ pack of the 3^rd^ region). Additionally, if available, these models may be informed by prior information on the diet composition of consumers. For instance, Moore and Semmens [15] used gut content data to develop priors for their analysis. It is important to note that the random effects models used in our analysis are computationally intensive and require a considerable amount of data in order to converge. Thus, while researchers with isotope data from 50 consumers will likely have success in fitting such multilevel models, researchers with data from 5 consumers will likely not. In order to facilitate the application of these models by other researchers, we have prepared supplemental material (Appendix S1) that includes: 1) a guide to simulating and fitting hierarchical variation in stable isotope data, 2) an exemplary problem with associated data, 3) detailed descriptions of the model likelihood calculations, and 4) a step-by-step explanation of the model code so that researchers can quickly and easily interpret and adapt these methods.

### Statistical Approach

Our approach extends the stable isotope mixing model discussed by Moore & Semmens [15]. Model parameters are the unobserved vector of diet proportions 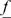, representing the relative contributions of *n* prey sources 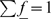. The sample mean and variances of the source and fractionation values are treated as known (*m*, 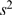), and used to estimate the means and variances of the mixture for each of *j* isotopes:
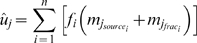
(1)

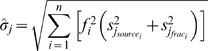
(2)Stable isotope data from multiple isotopes are then combined with the 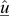 and 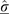 to evaluate the normal likelihood, with independence assumed between isotopes [15], [19].

We propose two new techniques for incorporating individual variation in Bayesian stable isotope mixing models. These approaches are generic, and may be incorporated into more complicated models that include multiple levels of nested variation (subpopulation, individual) or non-nested factors (sex, size class). The first method introduces variation in individual consumer diets by modeling diet proportions as a weighted mixture of individual and group effects using the Dirichlet distribution. A second, and potentially more flexible approach uses geometric transformations of 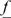, combined with random effects. These transformations normalize the compositional parameter space and thus afford the opportunity to apply standard general linear modeling methods.

Current tools for mixing models assume all consumers in the sampled population eat prey sources in the same relative proportions; in the Bayesian framework, the vector of prey contributions, 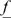, is assigned a Dirichlet prior distribution [15], [19]. One approach to incorporating niche variability into this model would be to treat the dietary proportions of each individual as independent Dirichlet distributions. Depending on the degree of individual variation, an alternative approach is to assume that a fraction of the diet proportions among all animals is the same, but the remaining portion of the diet is represented by individual variation. This latter approach involves modeling individual diet proportions using a weighted mixture of global and individual Dirichlet processes. We treat the single shared vector as 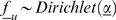, and each individual is allowed to have a unique vector of deviations, 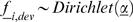. Diet proportions for each individual are then estimated as a weighted mixture, 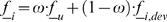, where ω can be modeled as a continuous (0,1) random variable. The weighted Dirichlet approach can be easily extended to include more than one level of hierarchical variation. To build a model of individuals nested within multiple geographic areas, we allow each area to have a unique mean, 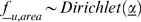. Individual deviations within each area are weighted by the area-specific mean, rather than the global mean (in both examples, all individuals share a single value of ω).

Our second technique for incorporating variability in diets among individuals is to use geometric transformations for compositional data, which have been widely used in the geosciences [20]. Stable isotope mixing models transform data from stable isotope δ-space to compositional diet *p*-space [11]; we build on previous work by applying geometric transformations to compositional diet proportions. The advantages of using these transformations are that additional sources of variation may be easily incorporated, and parameter estimation may be improved. Proposed transformations include the additive, centered, and isometric log-ratio transforms (ALR, CLR, ILR, respectively). We focus on the CLR transformation because it is isometric and treats components symmetrically [20], [21] and because it is numerically tractable in the mixing model framework (in contrast, the ILR involves solving polynomial roots). To illustrate a simple example, consider the basic stable isotope mixing model with no individual variation [15], where the estimated parameters are the vector of proportions 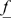 for *n* prey items. Instead of estimating 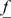 directly, an equivalent approach involves the CLR transformation, 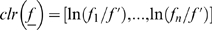, where the proportions are centered by the geometric mean, 
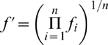
 [21].

As an example, consider a 2-isotope mixing model with a population of consumers that differ individually in their consumption of 4 prey. In CLR transformed space, there are 4 means, 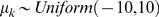, *k* = 1∶4. Alternatively, the 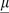 may be assigned Normal priors (with equal or different variances), or if enough data exist, the means may be jointly assigned a multivariate Normal distribution. The deviations of each individual are treated as random effects around the global mean (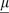). At the simplest level, deviations for each animal are univariate Normal with a single variance, 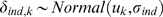. Even in data-limited situations, the assumption of a single variance term across diet components is reasonable when using the CLR transformation because dividing by the geometric mean places diet components on similar scales. With more samples, each of the transformed variables may be allowed to have a unique variance. Alternatively, a multivariate approach may be used, 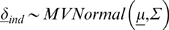.

Models with random effects may be easily extended to include multiple levels of variation [22]. Assume that in addition to individual variation, there is biological justification for including variation among several geographic areas. In this context, the global mean (shared between all individuals in all areas) is still assigned a uniform distribution, 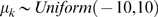, *k* = 1∶4. Area-specific deviations are treated as random effects centered around the global mean 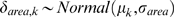 and individual deviations are centered around area specific means, 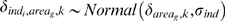. While it may be possible to share variance parameters 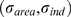 among levels, doing so prevents quantifying the relative magnitude of each type of variation, which may be useful in determining how niches vary by scale.

### Hierarchical Models of Wolf Diets in Coastal British Columbia

To illustrate the applicability of these hierarchical mixing models, we analyzed stable isotope data collected from a gray wolf population from coastal British Columbia, Canada. These wolves consume both terrestrial and marine prey, with the latter showing elevated carbon and nitrogen isotope signatures compared with terrestrial foods [23]. These data provide the opportunity to estimate the contribution of prey with dissimilar isotopic signatures to consumers while accounting for niche variation at multiple levels of population organization. The wolves predominantly consume three prey groups (deer, salmon, and marine mammals; [14], [24], [25]). Carbon and nitrogen stable isotope signatures were estimated from hair samples from 64 wolves, collected over four years (2001–2004). A more detailed description of these data, including how wolves and prey were sampled and estimates of fractionation were applied, is described by Darimont et al. [14]. Individuals from three subpopulations (distinct geographic areas) were represented in the samples: mainland, inner islands (adjacent to the mainland), and outer islands. Within these subpopulations, individuals are organized into known social groups (our analysis included data from social groups with at least four sampled individuals). Accordingly, we reasoned that the subpopulation, social group, and individual levels might all contribute to variation in estimated diet across the population.

We applied both the CLR and Dirichlet mixture methods described above to 8 different hierarchical mixing models. The simplest parameterizations we considered used a single invariant diet shared between all individuals [15] and the extension of this same model that includes residual error terms on each isotope [19]. Residual error accounts for generic, normally distributed variability in consumer isotope signatures beyond that explained by the basic mixing model formulation (equations 1–2); this error parameterization is thus largely phenomenological since it captures variability in the isotope data, but not variability in the diet of the consumer. Because we expected the geographic isolation and ecological context of each subpopulation to play a large role in shaping niche variation among individuals [14], we considered models with regional variation alone, pack variation nested within region, and regional variation in diet with residual errors on the consumer isotope signatures. Following Bolnick et al. [6], [26], we expected individual variation to play a potentially large role in shaping niche widths of populations. Accordingly, three models were constructed to allow for individual variation: individual variation alone, individual variation nested within region (no variation among packs within a region), and a 3-level model nesting individual variation within groups and group variation within each subpopulation. Support for Van Valen's [5] niche variation hypothesis was evaluated by comparing models that allowed variance parameters to vary spatially (by region) to models that shared variance parameters among regions. This allowed us to evaluate whether an area with larger sub-population niche width also showed greater inter-individual variation. For this last analysis, wolves from outer islands and inner islands were combined because of small sample sizes on outer islands.

### Parameter Estimation and Model Selection

For the Dirichlet models, hyperparameters were chosen to be non-informative (α = 1) and the mixture parameter ω was assigned a Uniform(0, 1) distribution. For all CLR models, uniform priors were assigned to the highest level mean in the model (global or region), and all lower level deviations were treated as independent normal random variables. Uniform priors were assigned to the standard deviation of all levels of random effects [27], and to the standard deviation of 6residual error [19]. We used the Deviance Information Criterion (DIC, [28]) to evaluate which models were most supported by the data. Gibbs sampling was performed for each model using 3 parallel chains in JAGS [29]. Following a burn-in phase of 5000 vectors, we sampled 50000 remaining vectors (retaining every 10^th^ sample) [30]. Convergence and diagnostic statistics were performed using the CODA package [31]. Diagnostics for the best model, and open source code for all interested readers (including R code to simulate data) is provided at http://www.ecologybox.org.

## Results

**Figure 1:**
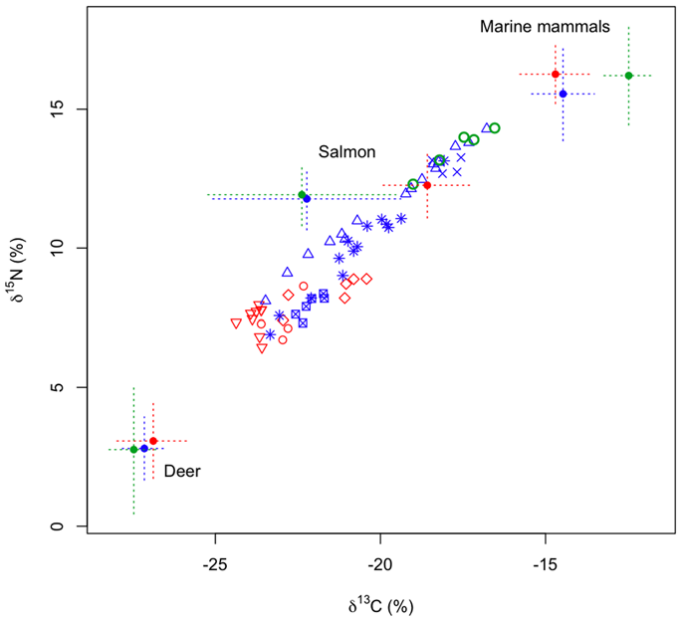
Stable isotope inputs to the hierarchical mixing model for B.C. wolves. Data derived from three regions (mainland, inner islands adjacent to the coast, outer islands). Prey items from each region have unique means (solid dots) and standard deviations (dashed lines) in each isotope dimension. For wolves (n = 64), symbols are used to depict group (pack) membership.

**Figure 2:**
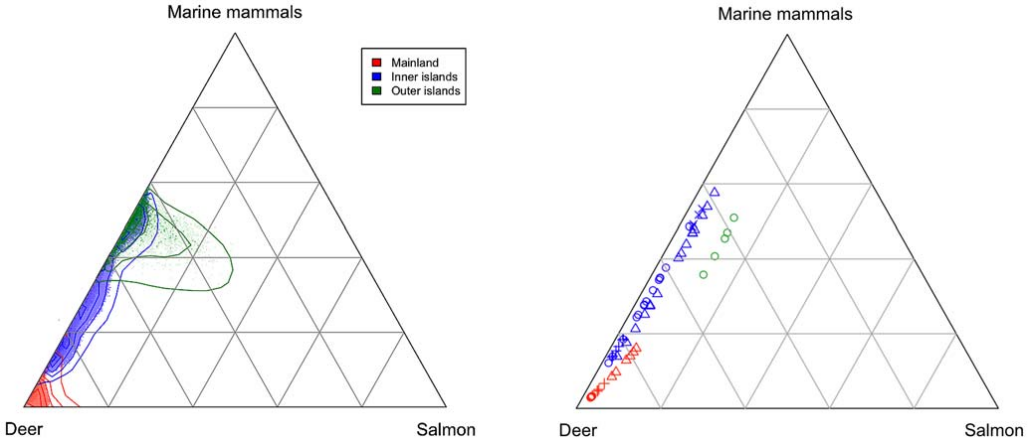
Ternary plots of posterior estimates of the proportional contribution of three prey types to the diet of wolves. Shown are posteriors for each region (aggregated across individuals) and medians (symbols denote group membership for individual wolves).

**Figure 3:**
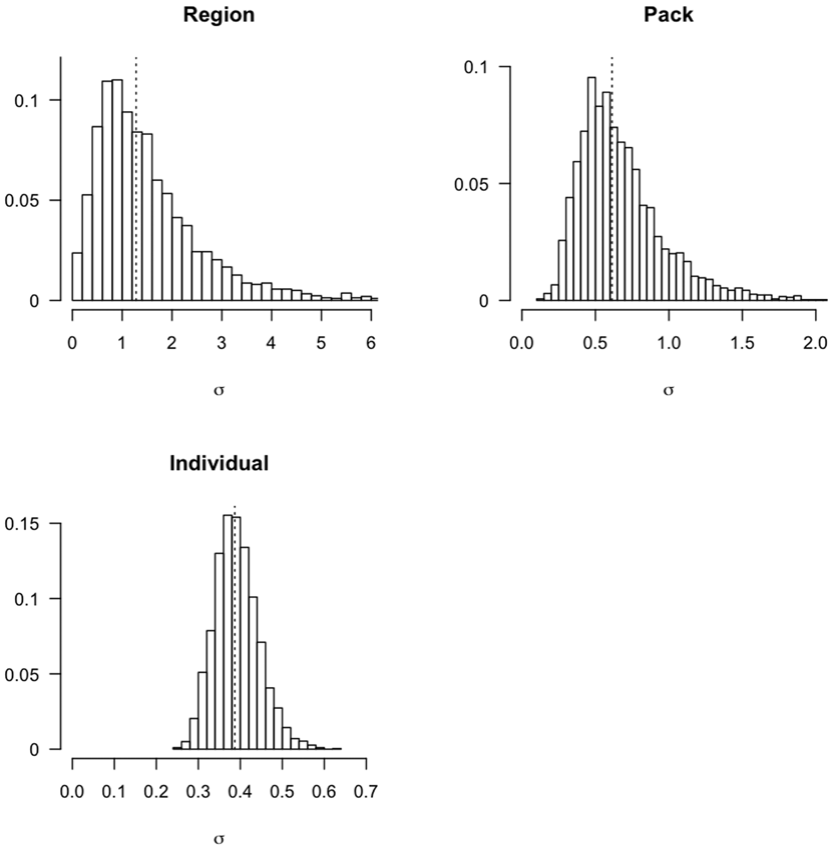
Estimated posterior density for the standard deviation parameters controlling the variation in diet across three scales (sub-population, social group, individual). Posterior densities are estimated from the model with the lowest DIC value, with medians indicated by dashed lines.

Wolves of coastal BC showed considerable intrapopulation variation in trophic niche, which was expressed at multiple levels of population structure. There was little support for models of wolf diet that did not include regional variation. For the single-level models without individual variability, including residual error terms on isotopes improved model fit substantially (models 2,4; Table 1). Similarly, residual error terms improved the fit for the model that included only regional differences in diet, but no variability in pack or individual diets. These results were likely due to the high variability in consumer isotope signatures relative to prey (Fig. 1). However, models with residual error ranked lower than any of the models that partitioned some of the total variance to the pack or individual levels (models 5,7,8; Table 1). The model most supported by the data (*i.e.* lowest DIC score) was one that included three hierarchical components of variation: at the regional, pack, and individual levels (model 8, Table 1, Fig. 2). While data strongly supported including individual variation, the posterior median of estimated variability among individuals (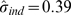) was smaller than the among-pack variability (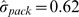
_)_, and both individual and pack variation were much smaller than regional variation (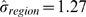, Fig. 3).

**Table 1:** Summary of results for 8 stable isotope mixing models explaining variation in diet for 64 wolves in British Columbia.

	Dirichlet					CLR				
Model	Region	Pack	Individual	Residual	DIC	Region	Pack	Individual	Residual	DIC
1	N	N	N	N	1142.30	N	N	N	N	1342.74
2	N	N	N	Y	586.20	N	N	N	Y	585.05
3	Y	N	N	N	692.22	Y	N	N	N	693.10
4	Y	N	N	Y	512.40	Y	N	N	Y	512.12
5	Y	Y	N	N	501.16	Y	Y	N	N	502.21
6	N	N	Y	N	347.78	N	N	Y	N	334.57
7	Y	N	Y	N	338.91	Y	N	Y	N	332.51
8	Y	Y	Y	N	NA	Y	Y	Y	N	325.56

Models may include variation among regions, packs (social groups), individuals, or residual error. The Deviance Information Criterion is used to evaluated data support, with smaller values signaling stronger support from the data. Two approaches to dealing with compositional data (Dirichlet mixtures or CLR transformed data) yielded similar results (NAs represent models with convergence issues).

**Figure 4:**
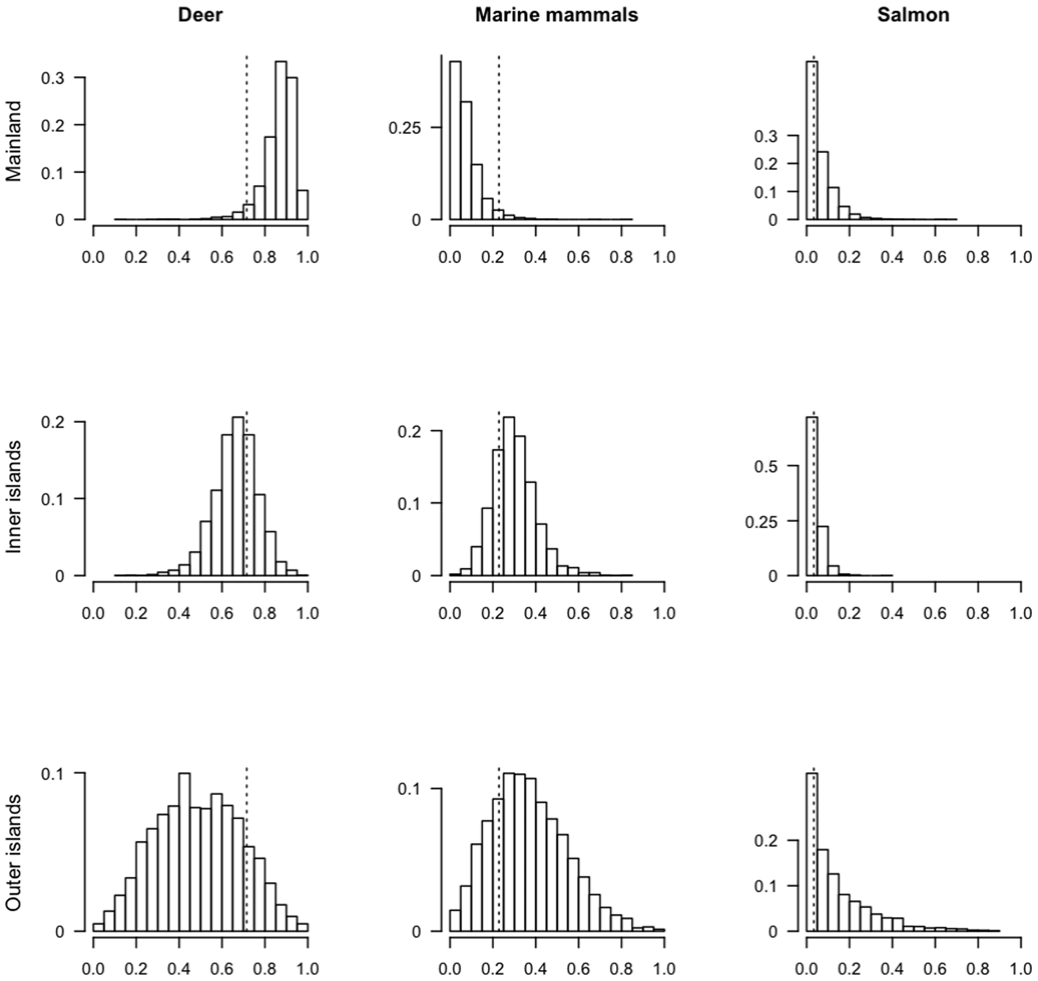
Region-specific posterior contributions of three prey items consumed by coastal wolf populations (deer, marine mammals, salmon). Posterior densities are drawn from the model with the lowest DIC value. Dashed lines depict the global median across three regions (mainland, inner islands adjacent to the coast, outer islands).

Based on the magnitudes of the variance parameters, the majority of the total variation in the diets of British Columbia wolves was driven by geographic region. Accordingly, we express dietary composition data at this scale. For the mainland subpopulation, median posterior estimates indicated deer represented the largest proportion of the diet (∼88%), while salmon (∼4%) and marine mammals (∼7%) represented only modest dietary proportions (Fig. 4). For the inner island subpopulation, there was a dramatic shift to increased use of oceanic prey; approximately 24% of the diet from deer was replaced by salmon and marine mammals (Fig. 4). Outer island wolves appeared to consume even more marine resources, with salmon and marine mammals contributing a combined 43% of the diet (Fig. 4, Table 2).

**Table 2:** Posterior estimates of diet proportions by region (subpopulation) for a coastal grey wolf population in British Columbia (n = 64).

Subpopulation	Deer	Marine mammals	Salmon
Mainland (n = 19)	0.882 (0.075) [0.670–0.959]	0.071 (0.070) [0.008–0.268]	0.035 (0.046) [0.001–0.162]
Inner islands (n = 40)	0.672 (0.101) [0.448–0.849]	0.291 (0.098) [0.124–0.513]	0.030 (0.033) [0.001–0.118]
Outer islands (n = 5)	0.527 (0.193) [0.153–0.874]	0.333 (0.169) [0.071–0.720]	0.075 (0.135) [0.001–0.499]

The median estimates of each prey item are given along with standard errors (parentheses) and 95% posterior intervals. All summary statistics are all generated from Figure 4.

Three models were compared to evaluate support for Van Valen's [5] niche variation hypothesis: we compared the best model with a shared individual variance across regions (Table 1, Model 8, DIC = 325.6) to models that assigned different pack level variance or individual level variance to wolves from islands (both inner and outer) versus wolves on the mainland. Each of these models introduced one additional parameter. While there was little support for allowing differences in group level variances (DIC = 325.7), there was strong support for a model allowing mainland and island wolves to have different levels of individual variability from the pack mean (DIC = 322.2). This latter model estimated variation among individuals on islands to be larger than variation among individual mainland wolves (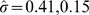). Thus, islands wolves, which had wider sub-population niche width, also showed greater among-individual variation.

## Discussion

The evolution of stable isotope analyses continues to yield powerful tools for inferring trophic ecology based on the chemical composition of consumers and prey. Our modeling approach is unique in that it can simultaneously estimate not only the composition of a population's diet but also the variation in diet among several nested components of the population. This integrated analytic framework improves the ability of mixing models to account for dietary variability, and the ability of isotope niche width analysis to directly assess the trophic links of a population. We applied this modeling approach to a coastal population of gray wolves with multiple levels of population structure (e.g., individual, pack, region), and found that individual dietary variability drives niche width expansion on islands.

Quantifying inter-individual niche variation can play a critical role in understanding a population's niche width [6]. Previous studies have typically relied on measures of proportional similarity among diets of individuals as a proxy for variance [32], [33]. Bolnick et al. [6] and Bearhop et al. [13] proposed that stable isotopes can be used to quantify specialization by comparing the variability in individual isotopes to the total isotopic variability of the population. However, consumer isotope variability is influenced both by individual differences in consumer diet and the variation in isotope signatures of prey items [34]. Thus, comparisons of niche width across study groups (e.g., populations) based on isotope variability may be confounded by differences in the isotope variability of their respective prey resources. Araújo et al. [12] developed a methodology that uses dietary data to construct a null model of niche width against which observed carbon isotope variability is compared, thus incorporating both prey and predator isotope signatures. However, this method depends on corresponding dietary information and also is constrained to the use of one isotope without fractionation. Newsome et al. [11] suggested using the products of isotopic linear mixing models (i.e., estimates of proportional contributions of prey) to calculate intra- and inter-population niche variability. However, deterministic mixing models do not incorporate potentially large sources of uncertainty such as too many sources, variation in fractionation, and prey isotope signatures [15].

Here we build on these previous methods by introducing two mechanistic approaches (Dirichlet mixture and CLR transform) to modeling individual variability within a hierarchical Bayesian mixing model framework. By modeling variation in diet across levels of population structure (e.g., individual, group, population), these approaches offer the ability to quantify the niche width of a consumer based on explicit estimates of the variability in source contributions to diet, as opposed to implicitly assuming that the variability in the isotope signatures of consumers directly reflects variability in diet. Estimating these additional levels of diet variation necessarily increases model complexity, requiring more careful consideration of data support. Data support for alternative levels of variation may be evaluated by comparing a suite of alternative models, as illustrated in our analysis of gray wolf data.

We anticipate that the approaches we outline here will assist in the application of isotopes to characterizing dietary variability within and between populations. While the quantitative methods we have outlined build upon previous efforts, they are still limited by basic assumption inherent to isotope mixing models [35], [36]. For example, we have assumed that prey sources are known and their isotopic signatures are quantified on an appropriate temporal and spatial scale [37], that prey sources are distinct enough to allow for source partitioning [13], that there is no concentration dependence [38] or tissue compartmentalization in mixing processes [35], that isotopic fractionation values are correctly quantified [39] and fractionation does not vary across populations [34]. While these assumptions are standard in mixing models, violations can influence model results. Both the Dirichlet mixture and CLR transform approaches appeared to work equally well (Table 1). However, because the CLR transform offers a more straightforward (linear) implementation, we anticipate future mixing model applications will rely principally on this approach. We note here that the CLR transform method can be used in mixing models with or without individual variability.

Using the same isotope data we analyzed, Darimont et al. [14] also found evidence supporting the niche variation hypothesis (estimated using isotopic variability, island wolves exhibited both the largest niche width and greatest among-individual variation; Fig. 1). The key difference, however, between the approach in Darimont et al. [14] and the hierarchical mixing model presented here is that the latter explicitly evaluates niche width based on diet proportion variability (estimated from a mixing model), rather that niche width based on isotope variability as a proxy for diet proportion variability. This is an important distinction because consumers in different regions may consume prey with more or less isotopic variation. In other words, it is possible that consumers on islands had broader isotopic variability due to the broader isotope variability of prey, and not because their diets were more variable.

**Figure 5:**
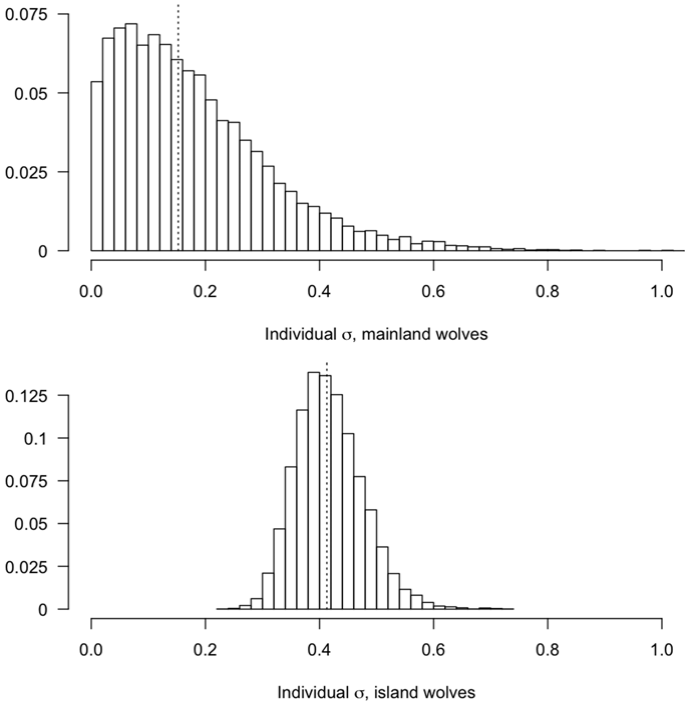
Posterior distributions of variation in individual diets for island and mainland wolves (median represented with dashed line). The variability for each region represents the deviation in diet from the social group (pack) diet.

Previous tests of Van Valen's [5] niche variation hypothesis have used morphological measurements or resource use [26]; our hierarchical models allow a quantitative evaluation of this hypothesis using mixing models with stable isotope data. Although a comparison of only two regions (mainland, island) is not a robust test of the niche variation hypothesis, we found additional and direct support for trophic niche expansion in wolves, with island wolves – that showed the larger niche width – also exhibiting more intra-group diet variability than wolves from the mainland (Fig. 5).

Understanding the relative contribution of different sources of variation in niche has important implications for community, evolutionary, and conservation ecology [5], [6], [25], [34], [40]–[44]. The mixing models we have detailed allow explicit quantification of dietary variability across multiple scales (population, group, individual) and diet estimates for each consumer. Because the model tools are highly flexible, they can be widely applied to any ecosystem, or more generally, to any ecological problem that relies on compositional data, structured hierarchically or not. For example, our approach would support examinations of niche variation as influenced by age [45], sex [46], morphology [47], genotype (*review in* [48]), and even cultural heritage [49]. Furthermore, the model selection framework employed here allows the explicit evaluation of model complexity based on data support. By incorporating dietary variability into the isotope mixing model framework, we have provided the tools necessary to assess the niche width of a consumer population based on variability in diet, rather than variability in isotope signatures. We anticipate that the application of these approaches will yield important advances in the application of isotope data in evolutionary ecology and conservation.

## Supporting Information

Appendix S1 Supporting documents to help researchers evaluate, interpret, and apply the modeling approaches used in this article.
(0.34 MB ZIP)

## References

[1] Chase JM, Leibold MA (2003). Ecological Niches: Linking Classical and Contemporary Approaches.

[2] Elton CS (1927). Animal Ecology.

[3] Leibold MA (1995). The niche concept revisited: mechanistic models and community context.. Ecology.

[4] Hutchinson GE (1957). Concluding Remarks..

[5] Van Valen L (1965). Morphological variation and width of ecological niche.. American Naturalist.

[6] Bolnick DI, Svanbäck R, Fordyce JA, Yang LH, Davis JM (2003). The ecology of individuals: incidence and implications of individual specialization.. American Naturalist.

[7] Giraldeau LA, Caraco T (2000). Social Foraging Theory.

[8] Goss-Custard JD, Clarke RT, Durell SEA Le V Dit (1984). Rates of food intake and aggression of oystercatchers Haematopus ostralegus on the most and least preferred mussel Mytilus edulis beds of the Exe estuary.. Journal of Animal Ecology.

[9] Radford AN, du Plessis MA (2003). Bill dimorphism and foraging niche partitioning in the green woodhoopoe.. Journal of Animal Ecology.

[10] Layman CA, Arrington DA, Montana CG, Post DM (2007). Can stable isotope ratios provide for community-wide measures of trophic structure?. Ecology.

[11] Newsome SD, Martinez del Rio C, Bearhop S, Philips DL (2007). A niche for isotopic ecology.. Frontiers in Ecology and the Environment.

[12] Araújo MS, Bolnick DI, Machado G, Giaretta AA, Reis SF (2007). Using δ^13^C stable isotopes to quantify individual-level diet variation.. Oecologia.

[13] Bearhop S, Adams CE, Waldron S, Fuller RA, MacLeod H (2004). Determining trophic niche width: a novel approach using stable isotope analysis.. Journal of Animal Ecology.

[14] Darimont CT, Paquet PC, Reimchen TE (2009). Landscape heterogeneity and marine subsidy generate extensive intrapopulation niche diversity in a large terrestrial vertebrate.. Journal of Animal Ecology.

[15] Moore JW, Semmens BX (2008). Incorporating uncertainty and prior information into stable isotope mixing models.. Ecology Letters.

[16] Fry B, Joern A, Parker PL (1978). Grasshopper Food Web Analysis: Use of Carbon Isotope Ratios to Examine Feeding Relationships Among Terrestrial Herbivores.. Ecology.

[17] Lubetkin SC, Simenstad CA (2004). Two multi-source mixing models using conservative tracers to estimate food web sources and pathways.. Journal of Applied Ecology.

[18] Phillips DL, Gregg JW (2001). Uncertainty in source partitioning using stable isotopes.. Oecologia.

[19] Jackson AL, Inger R, Bearhop S, Parnell A (2009). Erroneous behaviour of MixSIR, a recently published Bayesian isotope mixing model: a discussion of Moore & Semmens (2008).. Ecology Letters.

[20] Aitchison J (1986). The Statistical Analysis of Compositional Data Monographs on Statistics and Applied Probability.

[21] Egozcue JJ, Pawlowsky-Glahn V, Buccianti A, Mateu-Figueras G, Pawlowsky-Glahn V (2006). Simplical geometry for compositional data.. Compositional Data Analysis in the Geosciences: From Theory to Practice.

[22] Gelman A, Hill J (2006). Data Analysis Using Regression and Multilevel/Hierarchical Models.

[23] Schoeninger MJ, Deniro MJ (1984). Nitrogen and carbon isotopic composition of bone collagen from marine and terrestrial animals.. Geochimica et Cosmochimica Acta.

[24] Darimont CT, Price MHH, Winchester NN, Gordon-Walker J, Paquet PC (2004). Predators in natural fragments: foraging ecology of wolves in British Columbia's Central and North Coast Archipelago.. Journal of Biogeography.

[25] Darimont CT, Paquet PC, Reimchen TE (2007). Stable isotopic niche predicts fitness of prey in a wolf-deer system.. Biological Journal of the Linnean Society.

[26] Bolnick DI, Svanback R, Araujo MS, Persson L (2007). Comparative support for the niche variation hypothesis that more generalized populations also are more heterogeneous.. Proceedings of the National Academy of Sciences of the United States of America.

[27] Gelman A (2006). Prior distributions for variance parameters in hierarchical models.. Bayesian Analysis.

[28] Spiegelhalter DJ, Best NG, Carlin BR, van der Linde A (2002). Bayesian measures of model complexity and fit.. Journal of the Royal Statistical Society Series B-Statistical Methodology.

[29] Plummer M (2003). JAGS: A program for analysis of Bayesian graphical models using Gibbs sampling; Proceedings of the 3rd International Workshop on Distributed Statistical Computing; Vienna, Austria..

[30] Gelman A, Carlin JB, Stern HS, Rubin DB (2004). Bayesian Data Analysis.

[31] Best NG, Cowles MK, Vines SK (1995). CODA Manual version 0.30.

[32] Bolnick DI, Yang LH, Fordyce JA, Davis JM, Svanbäck R (2002). Measuring individual-level resource specialization.. Ecology.

[33] Roughgarden J (1979). Theory of Population Genetics and Evolutionary Ecology: An Introduction.

[34] Matthews B, Mazumder A (2004). A critical evaluation of intrapopulation variation of d 13C and isotopic evidence of individual specialization.. Oecologia.

[35] Wolf N, Carleton SA, Martinez del Rio C (2009). Ten years of experimental animal isotopic ecology.. Functional Ecology.

[36] Martinez del Rio C, Wolf N, Carleton SA, Gannes LZ (2009). Isotopic ecology ten years after a call for more laboratory experiments.. Biological Reviews.

[37] O'Reilly CM, Hecky RE, Cohen AS, Plisnier PD (2002). Interpreting stable isotopes in food webs: Recognizing the role of time averaging at different trophic levels.. Limnology and oceanography.

[38] Phillips DL, Koch PL (2002). Incorporating concentration dependence in stable isotope mixing models.. Oecologia.

[39] Caut S, Angulo E, Courchamp F (2009). Variation in discrimination factors (Δ15N and Δ13C): the effect of diet isotopic values and applications for diet reconstruction.. Journal of Applied Ecology.

[40] Reimchen TE, Ingram T, Hansen SC (2008). Assessing niche differences of sex, armour and asymmetry phenotypes using stable isotope analyses in Haida Gwaii sticklebacks.. Behavior.

[41] Roughgarden J (1972). Evolution of niche width.. American Naturalist.

[42] Bolnick DI (2001). Intraspecific competition favours niche width expansion in Drosophila melanogaster.. Nature.

[43] Svanbäck R, Bolnick DI (2007). Intraspecific competition drives increased resource use diversity within a natural population.. Proceedings of the Royal Society of London B: Biological Sciences.

[44] Layman CA, Quattrochi JP, Peyer CM, Allgeier JE, Suding K (2007). Niche width collapse in a resilient top predator following ecosystem fragmentation.. Ecology Letters.

[45] Polis G (1984). Age structure component of niche width and intraspecific resource partitioning: can age groups function as ecological species?. American Naturalist.

[46] Shine R (1989). Ecological causes for the evolution of sexual dimorphism: A review of the evidence.. Quarterly Review of Biology.

[47] Price T (1987). Diet variation in a population of Darwin's finches.. Ecology.

[48] Jaenike J, Holt RD (1991). Genetic variation for habitat preference: evidence and explanations.. American Naturalist.

[49] Estes JA, Riedman ML, Staedler MM, Tinker MT, Lyon BE (2003). Individual variation in prey selection by sea otters: patterns, causes and implications.. Journal of Animal Ecology.

