# Supplementary material for: Quantifying Inter- and Intra-Population Niche Variability Using Hierarchical Bayesian Stable Isotope Mixing Models
Source: PLoS One. 2009 Jul 9;4(7):e6187. doi: 10.1371/journal.pone.0006187 (PMC2704373; doi:10.1371/journal.pone.0006187)
Supplement: Appendix S1 — Supporting documents to help researchers evaluate, interpret, and apply the modeling approaches used in this article. (0.34 MB ZIP) [file pone.0006187.s001.zip › Detailed_model_math.pdf]

Bayesian Mixing Model Overview

Regardless of which levels (population, individual, or neither) are included in the stable isotope mixing model, the diet proportions are always constrained to sum to unity, $\sum \underline{f} = 1$.

The estimated means and variances for each of *j* isotopes (given the mixture proportions $\underline{f}$), $\hat{u}_j = \sum_{i=1}^{n} \left[f_i \left(m_{j_{source_i}} + m_{j_{frac_i}} \right) \right]$, $\hat{\sigma}_j = \sqrt{\sum_{i=1}^{n} \left[f_i^2 \left(s^2_{j_{source_i}} + s^2_{j_{frac_i}} \right) \right]}$

are calculated given the known means and variances for each source $\left(m_{source}, s^2_{source} \right)$ and the known means and variances for fractionation value $\left(m_{frac}, s^2_{frac} \right)$. Fractionation values are generally considered to be shared across sources (e.g. this analysis, Moore & Semmens 2008).

As a hypothetical 2-source model, with samples collected from 2 regions, each isotope and each prey item is allowed to have unique isotopic means and standard deviations:

Isotope	Sample	Region	Data(x)	$m_{source1}$	$s_{source1}$	$m_{source2}$	$s_{source2}$	m_{frac}	s_{frac}
N	1	1	7.34	7.01	1.33	9.83	1.96	2.21	1.57
N	2	1	6.99	7.01	1.33	9.83	1.96	2.21	1.57

N	3	2	6.80	8.45	1.22	10.31	1.78	2.21	1.57
N	4	2	7.56	8.45	1.22	10.31	1.78	2.21	1.57
C	1	1	-28.86	-25.3	2.04	-22.7	2.43	0.45	1.11
C	2	1	-22.01	-25.3	2.04	-22.7	2.43	0.45	1.11
C	3	2	-26.32	-26.2	1.91	-21.03	1.69	0.45	1.11
C	4	2	-25.54	-26.2	1.91	-21.03	1.69	0.45	1.11

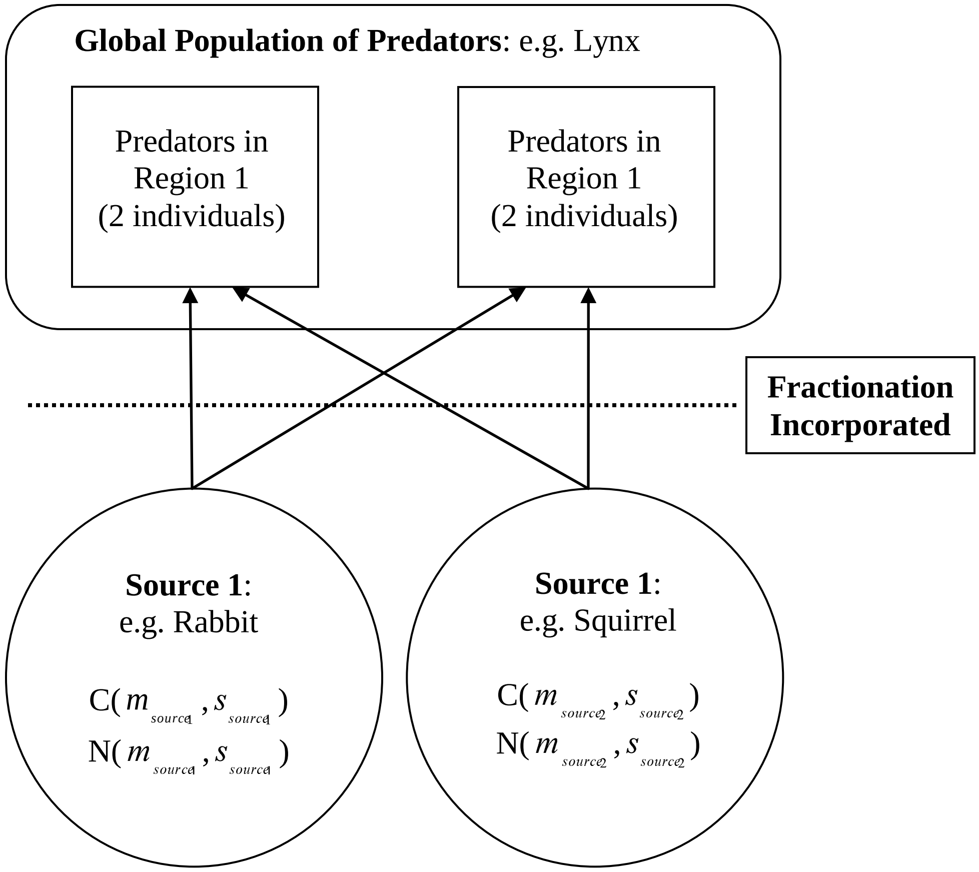

In this simple case, 4 individuals have been sampled (2 per region), and the source and fractionation values consumed by all individuals is assumed to be the same. Source values may also be stratified if there is variation in diets between strata (our analysis of wolf diets stratified sources by region, creating 3 vectors of region-specific means and standard deviations). A more complete discussion can be found in Moore & Semmens (2008).

CLR transformation

The centered log-ratio transformation 'centers' proportions by dividing each proportion by the total geometric mean,

$$\underline{f}_{clr} = \left[\ln(f_1 / f'),...,\ln(f_n / f')\right], \quad f' = \left(\prod_{i=1}^{n} f_i\right)^{1/n}$$

These transformed variables have two advantages: they are no longer constrained between 0 and 1, and no constraint is placed on their sum. After parameter estimation is complete, the CLR-transformed variables may be converted back to proportion space so that they may be interpreted biologically. This inverse transformation reimposes the sum to unity,

$$\underline{f} = \exp(\underline{f}_{clr}) / \sum \exp(\underline{f}_{clr})$$

Likelihood

The likelihood for the Bayesian stable isotope mixing model with no hierarchical variation is

$$L\left(\underline{x} \mid \underline{f}, \underline{\hat{u}}, \underline{\hat{\sigma}}\right) = \prod_{k=1}^{n}\prod_{j}\left(\frac{1}{\hat{\sigma}_j\sqrt{2\pi}}\exp\left[\frac{\left(x_{kj}-\hat{u}_j\right)^2}{2\hat{\sigma}_j^2}\right]\right) \qquad [1]$$

where x represents data (stable isotope measurements of consumer samples), j indexes isotopes and k indexes samples (Moore & Semmens 2008). The CLR transformation could be applied to $\underline{f}$, and the likelihoods would be equivalent: $L(\underline{x} \mid \underline{f}_{clr}, \underline{\hat{u}}, \underline{\hat{\sigma}}) = L(\underline{x} \mid \underline{f}, \underline{\hat{u}}, \underline{\hat{\sigma}})$.

Next, we can extend the model to include a single level of variation in populations. The simplest approach to modeling this variation is to assume that each level consists of normally distributed random effects. If we wanted to allow only population-level variation to be included, we would model the mth CLR-transformed diet proportion of each population as being distributed around a global mean, $f_{clr_m} \sim Normal(u_{pop_m}, \sigma_{pop})$. The dimension of $\underline{f}_{clr}$ (previously a vector with length $n_{sources}$) becomes a matrix with dimensions equal to $n_{sources} \times n_{populations}$. In this example, the CLR-transformed diet proportion of each source shares a unique global mean across populations, and all CLR-transformed proportions are assumed to share the common standard deviation (this latter assumption may be relaxed to allow source-specific variance parameters). The likelihood for all populations and sources can be written as

$$L(\underline{f}_{clr} \mid \underline{u}_{pop}, \sigma_{pop}) = \prod_{i=1}^{n_{populations}}\prod_{j=1}^{n_{source}}\left(\frac{1}{\sigma_{pop}\sqrt{2\pi}}\exp\left[\frac{(f_{clr_j}-u_j)^2}{2\sigma_{pop}^2}\right]\right) \qquad [2]$$

The total likelihood function for this model is then the product of [1] and [2], $L(\underline{x} \mid \underline{f}_{clr}, \underline{u}_{pop}, \underline{\sigma}_{pop}) = L(\underline{x} \mid \underline{f}_{clr})L(\underline{f}_{clr} \mid \underline{u}_{pop}, \underline{\sigma}_{pop})$

[3]

The product of these two likelihoods is often separated out in the literature, with the first component given the name 'likelihood' and the second component referred to as the 'process model' (e.g. Clark 2005, *Ecology Letters*). Because the transformed diet proportions $\underline{f}_{clr}$ are not directly measureable, they may be referred to by a number of names, including latent variables, and random effects. For a complete Bayesian analysis, priors must be specified on each of the model parameters. We use a uniform prior for the standard deviation and independent uniform priors on each of the source means in CLR-space. The product of this prior and the likelihood results in the joint posterior,

$$P(\underline{f}_{clr}, \sigma_{pop}, \underline{u}_{pop} \mid \underline{x}) \propto L(\underline{x} \mid \underline{f}_{clr}, \underline{u}_{pop}, \underline{\sigma}_{pop})\pi(\sigma_{pop}, \underline{u}_{pop})$$

The single level model described above can be easily extended to include any number of hierarchical levels. For example, suppose we wish to include variation among social groups (nested within populations) and variation among individuals (nested within social groups). For each of *m* sources, we assume population means to be centered around a global mean [4], social group means to be centered around the population mean [5], and individual means to be centered around the group mean [6]:

$$u_{pop_m} \sim Normal(u_m, \sigma_{pop}) \qquad [4]$$

$$u_{group_m} \sim Normal(u_{pop_m}, \sigma_{group}) \qquad [5]$$

$$f_{clr_m} \sim Normal(u_{group_m}, \sigma_{individual}) \qquad [6]$$

The total likelihood from [3] can be written as

$$L(\underline{x} \mid \underline{f}_{clr}, \underline{u}_{pop}, \underline{\sigma}_{pop}) = L(\underline{x} \mid \underline{f}_{clr})L(\underline{f}_{clr} \mid \underline{u}_{group}, \underline{\sigma}_{individual})L(\underline{u}_{group} \mid \underline{u}_{pop}, \underline{\sigma}_{group})L(\underline{u}_{pop} \mid \underline{u}, \underline{\sigma}_{pop})$$

References

Clark, J.S., Why environmental scientists are becoming Bayesians, Ecology Letters, 8:2-14, 2005.
