# Supplementary material for: Quantifying Inter- and Intra-Population Niche Variability Using Hierarchical Bayesian Stable Isotope Mixing Models
Source: PLoS One. 2009 Jul 9;4(7):e6187. doi: 10.1371/journal.pone.0006187 (PMC2704373; doi:10.1371/journal.pone.0006187)
Supplement: Appendix S1 — Supporting documents to help researchers evaluate, interpret, and apply the modeling approaches used in this article. (0.34 MB ZIP) [file pone.0006187.s001.zip › Simulation_and_Model_Run_Walkthrough.pdf]

Simulating and Fitting Hierarchical Variation in Stable Isotope Data

E.J. Ward and B.X. Semmens

June 8, 2009

1 Generating source signatures

The first step in simulating hierarchical stable isotope data for Bayesian mixing models is to define the number of sources (prey items) and the limits on the source means and variances. Alternatively, these could be fixed at specific values. For this example, we'll start with 3 sources.

```
> src_num = 3
```

We'll assume that there are 2 isotopes (Carbon, Nitrogen). For each isotope, we'll specify the limits on the means and variances. Each source item will have a unique Carbon and Nitrogen signature (with unique standard deviations) - for example, salmon and deer in our wolf diet data have different means and standard deviations. For simplicity, we'll assume that all regions have the same source values, and we'll draw the means and standard deviations from uniform distributions within our specified limits:

```
> Cmax = -15
> Cmin = -30
> CmaxSD = 2
> CminSD = 1
> Nmax = 15
> Nmin = 0
> NmaxSD = 2
> NminSD = 1
> src_C <- rep(0, src_num)
> src_Csd <- rep(0, src_num)
> src_N <- rep(0, src_num)
> src_Nsd <- rep(0, src_num)
> for (i in 1:src_num) {
+     src_C[i] = runif(1, min = 0, max = 1) * (Cmax - Cmin) + Cmin
+     src_Csd[i] = runif(1, min = 0, max = 1) * (CmaxSD - CminSD) + CminSD
+     src_N[i] = runif(1, min = 0, max = 1) * (Nmax - Nmin) + Nmin
+     src_Nsd[i] = runif(1, min = 0, max = 1) * (NmaxSD - NminSD) + NminSD
+ }
```

Let's look at these randomly generated means for Carbon:

```
> src_C

[1] -25.58096 -18.29430 -23.02519
```

2 Generating regional variation

With these source values specified for each source, we can now specify the number of regions (or populations). For this example, we'll model 3 regions here.

```
> numregion = 3
```

If we want to include variation among regions, we need to specify the global mean diet (across all regions). These values have been arbitrarily chosen.

```
> # Alternatively,global.mu could be drawn from a Dirichlet distribution
> global.mu = c(0.05, 0.2, 0.75)
```

One approach to modeling the regional variation from the global mean is to use the 'compositions' library to use the CLR transformation. We transform the compositional data with CLR because it allows us to include level-specific random error subject to the constrains of proportional data (must sum to unity). We'll specify a regional standard deviation parameter, shared across regions and sources.

```
> library(compositions)
> clr.mu = clr(global.mu)[1:3]
> region.clr = matrix(0, nrow = numregion, ncol = src_num)
> region.p = matrix(0, nrow = numregion, ncol = src_num)
> region.sd = 0.7
> for (i in 1:numregion) {
+     region.clr[i, ] = rnorm(src_num, clr.mu, sd = region.sd)
+     region.p[i, ] = clrInv(region.clr[i, ])
+ }
> colnames(region.p) = paste("Prey ", 1:src_num)
> row.names(region.p) = paste("Region ", 1:numregion)
> region.p
```

```
             Prey  1   Prey  2   Prey  3
Region  1 0.19597637 0.1752958 0.6287278
Region  2 0.06061059 0.1124972 0.8268922
Region  3 0.15560793 0.1650175 0.6793746
```

3 Generating group variation within region

Next, we'll incorporate group or subpopulation variability from the regional means. We'll allow 3 groups per region, again with a common standard deviation shared between sources and groups (creating a total of nine groups).

```
> # Alternatively,global.mu could be drawn from a Dirichlet distribution
> group2region = rep(seq(1,numregion),3)
> numgroups = length(group2region)
> # group.clr holds the group means in CLR space
> group.clr = matrix(0,nrow=numgroups,ncol=src_num)
> # group.p holds the diet proportions for each group in normal space
> group.p = matrix(0,nrow=numgroups,ncol=src_num)
> group.sd = 0.49 # arbitrary standard deviation
> for(i in 1:numgroups) {
+    group.clr[i,] = rnorm(src_num, region.clr[group2region[i],], sd = group.sd)
+    group.p[i,] = clrInv(group.clr[i,])
+ }
```

```
> colnames(group.p)=paste("Prey ", 1:src_num)
> row.names(group.p)=paste("Group ", 1:numgroups)
> group.p

             Prey  1    Prey  2   Prey  3
Group  1 0.15554020 0.19477642 0.6496834
Group  2 0.02962156 0.07256560 0.8978128
Group  3 0.13583491 0.15070073 0.7134644
Group  4 0.14013612 0.08956242 0.7703015
Group  5 0.04049365 0.07055791 0.8889484
Group  6 0.07260589 0.17785528 0.7495388
Group  7 0.33032441 0.17940049 0.4902751
Group  8 0.07933489 0.12424395 0.7964212
Group  9 0.26052228 0.07385455 0.6656232
```

4 Generating individual variation within groups

The third component of nested hierarchical variation is individual variation nested within groups. We need to first define the number of individuals we want to simulate, and then assign each individual to a group (by doing so we also implictly assign individuals to regions).

```
> num_individuals = 60
> # randomly assign each to a group.  Alternative ways of assignment would be a fixed
> # number of equal or unequal individuals per group.
> ind2groups = sample(seq(1,numgroups), size = num_individuals, replace = T)
> ind2regions = group2region[ind2groups]
```

Once individuals are assigned, we can specify the individual deviation from the group mean, and generate the diet proportions for each animal.

```
> # ind.clr holds the individual means in CLR space
> ind.clr = matrix(0,nrow=num_individuals, ncol=src_num)
> # ind.p holds the diet proportions for each individual in normal space
> ind.p = matrix(0,nrow=num_individuals,ncol=src_num)
> ind.sd = 0.13 # arbitrary standard deviation
> for(i in 1:num_individuals) {
+    ind.clr[i,] = rnorm(src_num, group.clr[ind2groups[i],], sd = ind.sd)
+    ind.p[i,] = clrInv(ind.clr[i,])
+ }
```

5 Generating mixture distributions

Given the individual proportions and the source data (in Section 1 above), we can calculate the mean and the variance of the mixture for each isotope. These values represent the actual data (x) measured from each consumer.

```
> # mix.data will hold the stable isotope measurements for each animal
> mix.data = matrix(0, num_individuals, 2)
> # loop over individuals, calculating mixture for each
> for(i in 1:num_individuals) {
+    # loop over sources, because total measurement is a composite across sources
+    for(j in 1:src_num) {
+         # first is carbon, second is nitrogen
+         mix.data[i, 1] = mix.data[i, 1] + (ind.p[i,j] * (src_C[j]+src_Csd[j]*rnorm(1,0,1)))
+         mix.data[i, 2] = mix.data[i, 2]+ (ind.p[i,j] * (src_N[j]+src_Nsd[j]*rnorm(1,0,1)))
+    }
+ }
```

6 Estimating parameters (using JAGS)

A few things need to happen before we can estimate parameters. We choose to use JAGS, but you can also use OpenBUGS or WinBUGS to accomplish the same thing. Unlike WinBUGS or OpenBUGS, JAGS is really not platform specific (although OpenBUGS/WinBUGS can still be run on a mac with Wine or Parallels). Here are the steps:

1. Download and install JAGS for your platform, http://www-ice.iarc.fr/~martyn/software/jags/

2. From R, install some or all of the following libraries: runjags, R2WinBUGS, R2jags, BRugs, gtools, gdata

3. Set the MCMC parameters, as follows:

```
> mcmc.chainLength <- as.integer(50000)  # post-burn samples
> mcmc.burn <- as.integer(20000)
> mcmc.thin = 10;                 # thinning interval
> mcmc.adapt = 5000        # how long during the burn in should sampling be adaptive?
> mcmc.chains = 3              # number of MCMC chains
```

4. Define parameters and create a list of all the data we'll be passing to JAGS

```
> N = num_individuals
> num.prey = src_num
> num.pop = numregion
> num.iso = 2
> X = mix.data
> Group = ind2groups
> numGroup = numgroups
> Region = ind2regions
> groups2Region = group2region
> # create arrays to hold optional region specific means/variances
> u = array(0,dim=c(num.prey,num.iso,num.pop))
> sigma2 = array(0,dim=c(num.prey,num.iso,num.pop))
> # include values for fractionation...
> C13.frac.u = 2.1
> C13.frac.var = 0.22^2
```

```
> N15.frac.u = 3.1
> N15.frac.var = 0.178^2
> # Calculate the total mean and variance, incorporating fractionation
> for(j in 1:numregion) {
+    for(i in 1:num.prey) {
+       u[i,1,j]=src_C[i] + C13.frac.u
+       u[i,2,j]=src_N[i] + N15.frac.u
+       sigma2[i,1,j]=src_Csd[i]^2 + C13.frac.var
+       sigma2[i,2,j]=src_Nsd[i]^2 + N15.frac.var
+    }
+ }
> jags.data = list("u", "sigma2", "N", "num.prey", "num.iso", "num.pop",  "X","Group",
+ "numGroup","groups2Region","Region")
```

5. Generate initial values for the model run.

```
> jags.inits <- function(){
+ list("p.transform"=matrix(0,num.prey,num.pop),"p.group"=matrix(0,num.prey,numGroup),
+ "p.ind"=matrix(0,num.prey,N), "group.sig"=runif(1),"ind.sig"=runif(1),
+ "region.sig"=runif(1), "mu"=rep(0,num.pop))
+ }
```

6. Select the parameters to monitor (parameters not in this list won't be available to R).

```
> jags.params=c("p","group.sig","region.sig","ind.sig","mu")
```

7. Specify the name of the model (in this case, located in the same directory).

```
> model.loc="model.txt"
```

8. Run the JAGS model from the R prompt.

```
> library(R2jags)
> jags.model = jags(jags.data, inits = jags.inits, parameters.to.save= jags.params,
+ model.file=model.loc, n.chains = mcmc.chains, n.burnin = mcmc.burn, n.thin = mcmc.thin,
+ n.iter = mcmc.chainLength, DIC = TRUE, n.adapt = mcmc.adapt)
```

9. Summarize results

```
> summary(jags.model$BUGSoutput$sims.matrix)
```

10. Alternatives If you have a PC, there several GUI-based alternatives to running JAGS. You can download either OpenBUGS or WinBUGS - both programs allow the user to set preferences and sample parameters from within a GUI. To try these approaches, follow through step 4 above (to the command jags.data()). Next, we'll need to write the data file for OpenBUGS / WinBUGS. Install and load the "BRugs" library in R, and call the bugsData() function with a list of data (like we did with jags.data). Alternatively, you can call the bugs.data() function from the R2WinBUGS library - both approaches accomplish the same thing. When the data file is written, a new file ("data.txt") can be found in your current R directory - if you included all of the right variables, it is possible to load this data into WinBUGS / OpenBUGS.
