# Supplementary material for: Quantifying Inter- and Intra-Population Niche Variability Using Hierarchical Bayesian Stable Isotope Mixing Models
Source: PLoS One. 2009 Jul 9;4(7):e6187. doi: 10.1371/journal.pone.0006187 (PMC2704373; doi:10.1371/journal.pone.0006187)
Supplement: Appendix S1 — Supporting documents to help researchers evaluate, interpret, and apply the modeling approaches used in this article. (0.34 MB ZIP) [file pone.0006187.s001.zip › Stepping_through_BUGS_code.pdf]

Stepping through BUGS/JAGS code for estimating multilevel variation in stable isotope mixing models

E.J. Ward & B.X. Semmens
05/27/09

*All slides refer to model 8, available at
http://www.ecologybox.org (Individual Diet Modeling)

Establishing priors

```
for(prey in 1:num.prey) {
    mu[prey] ~ dunif(-3,5);
}

region.sig ~ dunif(0,20);
region.invSig2 <- 1/(region.sig*region.sig);

group.sig ~ dunif(0,20);
group.invSigma2 <- 1/(group.sig*group.sig);

ind.sig ~ dunif(0,20);
ind.invSigma2 <- 1/(ind.sig*ind.sig);
```

Prior on source-specific global CLR-transformed variables

Prior on regional variation

Prior on group variation

Prior on individual variation

Population variation from the global mean

```
for(pop in 1:num.pop) {
    for(prey in 1:num.prey) {
     p.transform[prey,pop] ~ dnorm(mu[prey],region.invSig2);
    }
}
```

CLR-transformed proportion for each population/source are independent normals with the same standard deviation

Group variation from the population mean

```
for(group in 1:numGroup) {
    for(prey in 1:num.prey) {
      p.group[prey,group] ~
    dnorm(p.transform[prey,groups2Region[group]],group.invSigma2);
    }
  }
```

CLR-transformed proportion for each group/source are independent normals with the same standard deviation

Individual variation from the group mean

```
for(i in 1:N) {
   for(prey in 1:num.prey) {
     # individual deviation from group mean
     p.ind[prey,i] ~ dnorm(p.group[prey,Group[i]], ind.invSigma2);
     exp.p[prey,i] <- exp(p.ind[prey,i]);
   }
 }
```

CLR-transformed proportion for each individual/source are independent normals with the same standard deviation

This bit is to get a head start on the inverse transformation from CLR-space to normal space (next slide)

Converting from CLR space to normal space

```
for(i in 1:N) {

    p.tot[i] <- sum(exp.p[1:num.prey,i]);

    for(prey in 1:(num.prey-1)) {
      p[prey,i] <- exp.p[prey,i]/p.tot[i];
    }

    p[num.prey,i] <- 1-sum(p[1:(num.prey-1),i]);

}
```

Loop over animals, calculating the denominator for the inverse CLR-transform

Calculate the diet proportions for all but the last source

Calculate the last diet proportion based on the unity sum constraint

Calculate weights for variances, p2 = p^2

```
for(prey in 1:num.prey) {
    for(i in 1:N) {
        # these are weights for variances
        p2[prey,i] <- p[prey,i]*p[prey,i];
    }
}
```

Calculate the predicted means/variances of the mixtures

```
for(iso in 1:num.iso) {

  for(i in 1:N) {

    mix.mu[iso,i] <- inprod(u[,iso,Region[i]],p[,i]);

    mix.totalVar[iso,i] <- inprod(sigma2[,iso,Region[i]],p2[,i]);
    # precision has to be calculated for the likelihood section
    mix.prcsn[iso,i] <- 1/(mix.totalVar[iso,i]);

  }

}
```

Mixture is for each isotope and is unique to each individual

Calculate the likelihood for all individuals given the estimated mixture mean/variance

```
for(i in 1:N) {

   for(iso in 1:num.iso) {
     X[i,iso] ~ dnorm(mix.mu[iso, i], mix.prcsn[iso, i]);
   }

}
```

Each individual has a unique mean and variance for each isotope, and these are assumed to be independent normal distributions
